# Successful management of a patient with a C3 Glomerulonephritis and crescentic pattern: a case report

**DOI:** 10.1186/1756-0500-7-792

**Published:** 2014-11-07

**Authors:** Ana Pinho, Graça Ferreira, Conceição Mota

**Affiliations:** Department of Nephrology, Centro Hospitalar Algarve, Faro Hospital, Leão Penedo, 8000-386 Faro, Portugal; Department of General Pediatrics, Centro Hospitalar Vila Nova de Gaia, Gaia, Portugal; Department of Pediatric Nephrology, Centro Hospitalar Porto, Porto, Portugal

**Keywords:** Alternative pathway of complement, C3 glomerulonephritis, Crescentic glomerulonephritis, Child

## Abstract

**Background:**

Crescentic glomerulonephritis is a rare condition in children and is typically associated with renal insufficiency. Dysfunction of the alternative complement pathway is an unusual aetiology with an unknown mechanism.

**Case presentation:**

We report a case of a previously healthy 12-year-old Caucasian girl who was examined on emergency owing to an asymptomatic gross haematuria. An active urinary sediment and nephrotic-range proteinuria were identified, and serologic examination showed a decreased serum C3 concentration not associated with any immunologic or infectious cause. Oedema, hypertension, and renal insufficiency were not observed. A renal biopsy was performed, and crescentic glomerulonephritis associated with C3 glomerulonephritis was diagnosed. Prompt treatment with intravenous steroids resulted in complete resolution of the gross haematuria. Further examination did not detect any underlying acquired cause. A combination of oral steroids and cyclophosphamide, followed by mycophenolate mofetil, was maintained and resulted in clinical remission during an 8-month follow-up.

**Conclusion:**

The presence of severe injury such as crescentic glomerulonephritis secondary to C3 glomerulonephritis is extremely unusual in children. This is the first known case of paediatric crescentic glomerulonephritis secondary to C3 glomerulonephritis that presented with gross haematuria and was treated early and effectively with immunosuppressive therapy based on its severe histologic features.

## Background

Crescentic glomerulonephritis (CsGN) is diagnosed in only approximately 5% of unselected renal biopsies in children and typically presents as rapidly progressive renal failure [1]. In developed countries, pauci-immune CsGN shows a similar or even higher incidence than does immune complex CsGN, which includes post-infectious GN [1–3]. Since updates to the glomerular classification of membranoproliferative GN, a new aetiology of CsGN has been described. CsGN is now thought to be caused by a different mechanism of glomerular injury, namely dysregulation in the complement alternative pathway (AP) [4]. Known as C3 glomerulopathy, recent reports suggest that it may be misdiagnosed as post-infectious GN [5]. C3 glomerulopathy may also cause ANCA-negative, pauci-immune CsGN [4]. Consequently, there is a great need to improve the differential diagnosis and understand the underlying pathology.

Herein, we report a paediatric case of CsGN secondary to dysregulation in the complement AP that presented with asymptomatic gross haematuria, was diagnosed early, and was treated effectively.

## Case presentation

A 12-year-old Caucasian girl with no notable medical history, except for allergic rhinitis, presented with asymptomatic gross haematuria lasting for 7 days. She had no systemic symptoms, sore throat, skin rash, or dysuria, and had no known recent infections. There was no family history of kidney disease. The initial examination showed a temperature of 37°C, blood pressure of 109/55 mmHg, pulse rate of 76 beats/min, and a respiration rate of 20 breaths/min. She had a stable body weight of 39.5 kg that was appropriate for her height of 149 cm. The cardiovascular, lung, and neurologic examinations were unremarkable. Urine output was maintained. The laboratory evaluation showed a normal blood cell count, normal serum protein and albumin concentrations, and normal renal (Cr 0.66 mg/dl) and lipid profiles. The urinalysis revealed an increased urine protein-creatinine ratio (uPCR) of 3580 mg/g and an active urinary sediment (erythrocytes >50/HCF, leukocytes 25–50/HCF, and red blood cell casts 2–5). Multiple serologic examinations, including an antistreptolysin O titer, antinuclear, glomerular basement membrane, and antineutrophil cytoplasmic antibodies, serum protein electrophoresis, cryoglobulins, hepatitis serologic tests, and human immunodeficiency virus, were normal or negative. A complement protein evaluation showed a decreased serum C3 (50.6 mg/dl [normal 81–167 mg/dl]) and normal C4 (26.4 mg/dl [normal 11–42 mg/dl]) concentrations. No infectious cause or other triggering event was detected, as blood and urine cultures were negative. An ultrasound examination revealed hyperechogenicity in the renal cortex bilaterally.

**Figure 1:**
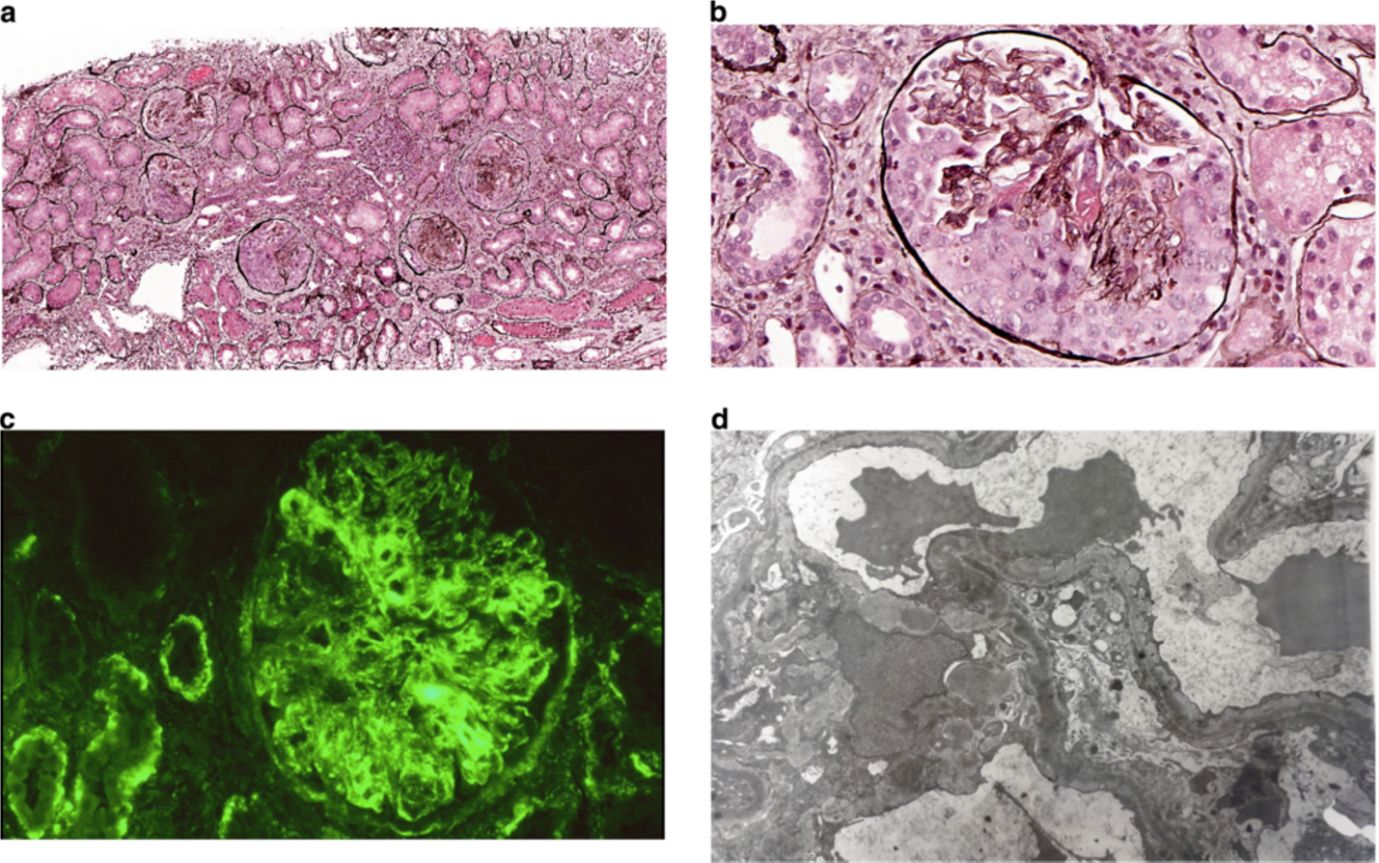
**Histopathologic examination of the kidney in a 12-year-old Caucasian girl with asymptomatic haematoproteinuria. a**. Low-power magnification of five glomeruli showing cellular crescents (Jones stain; original magnification, ×10). **b**. High-power magnification of one noncircumferential cellular crescent with rare intracapillary neutrophils (Jones stain; original magnification, ×40). **c**. Immunofluorescence showing granular deposits of C3 (+++) within the mesangium, glomerular capillary loops, and tubular basement membrane (original magnification, ×40). **d**. Electron microscopy showing intermediate electron-dense deposits within the glomerular capillary loops and mesangium (original magnification, ×40,000).

The C3 remained persistently decreased, and the patient failed to clinically improve over the subsequent 7 weeks; therefore, a renal biopsy was performed. Histopathologic examination of the tissue showed cellular crescent formations in more than 80% of 23 glomeruli that were evaluated (Figure 1a); the formations tended to be large but were not circumferential (Figure 1b). There was no interstitial inflammation, tubular atrophy, or interstitial fibrosis observed. The vessels were unremarkable. The mesangium along the capillary wall showed strong (+2) staining on immunofluorescent evaluation (Figure 1c). A C3 glomerulopathy was suspected and was clarified by electron microscopy. Ultrastructure examination revealed mesangial and paramesangial intermediate electron-dense deposits; sausage-shaped, large electron-dense deposits were not observed (Figure 1d).

Based on the characteristic pathology and the isolated dysfunction in AP activity, the patient was diagnosed with C3 glomerulonephritis (C3GN) with a CsGN histologic pattern.

Because of the crescentic pattern, the patient was initially administered intravenous methylprednisolone (1 g) three times on alternate days, followed by oral prednisolone (60 mg/day). The serum complement concentration subsequently normalised, and the gross haematuria ceased. Enalapril (at a maximally tolerated dose of 20 mg daily) and cyclophosphamide (intravenous bolus of 500 mg monthly) were administered over the next 6 months, and the corticosteroid dose was gradually tapered as the uPCR decreased. During this period, she experienced a single episode of gross haematuria, which promptly resolved with an increase in the corticosteroid dose. No additional episodes of decreased C3 or C4 was noted on serum examinations during treatment. However, immediately after discontinuing cyclophosphamide, the AP functional activity decreased to very low values (6.1%, [normal range 30%–113%]); this trend promptly reversed with the introduction of mycophenolate mofetil, and the AP functional activity increased to 85.4%. The circulating C3NeF was negative, the serum CFH concentration was 99.4 mg/dl (normal range 34.5–59 mg/dl), and the soluble membrane attack complex (sC5b9) was normal. The genetic analysis was negative.

Eight months after beginning treatment, the patient was well and asymptomatic. She remained stable as the prednisolone was tapered to 15 mg every other day along with 1.5 g of mycophenolate mofetil daily. She maintained normal renal function (Cr 0.4 mg/dl) and showed no signs of analytic AP dysfunction, though she continued to show a persistent mild haematoproteinuria (urine erythrocytes 5–10/HCF and uPCR 960 mg/g).

## Discussion

A crescentic pattern occurring simultaneously with C3GN is extremely unusual, and the present case appears to be the first known paediatric case presenting with an asymptomatic gross haematuria. There is one previous report describing a patient diagnosed with C3GN who presented with recurrent gross haematuria, underwent a renal biopsy, and developed nephrotic syndrome only 1.5 years later; the case is included in a case series of C3 glomerulopathy in children [6]. In that particular case, owing to the signs of severe chronic disease, the renal biopsy was delayed to prevent accelerating the development of end-stage renal disease. In the same report, the investigators also described three cases of CsGN (crescents >50%) associated with C3GN that initially presented with signs of acute renal failure [6]. In all three cases, normal kidney function was restored and the disease was controlled by the end of follow-up with timely immunosuppression treatment.

As the understanding of complement-mediated kidney disease has improved, a new classification of C3 glomerulopathy has emerged. Renal disorders are categorised based on the presence of glomerular C3 deposits in the absence of significant immunoglobulin deposition and independent of glomerular disease patterns [7]. The rationale of this classification scheme is to categorise glomerulopathies according to the pathogenetic mechanism instead of the histologic pattern in order to guide the clinical evaluation and provide disease-specific treatments [8]. These disorders, which are mediated by complement dysregulation leading to persistent, uncontrolled AP activation, can be further categorised into dense deposit disease (DDD) and C3GN [7–9]. Any presence of sausage-shaped, amorphous, dense deposits in the lamina densa of the glomerular basement membrane or similarly dense nodular mesangial deposits on electron microscopy indicates DDD rather than C3GN [9]. The present case showed predominantly C3 staining with no immunoglobulin deposition and an absence of dense deposits, favouring a diagnosis of C3GN.

A diagnosis of C3GN is suggested by the presence of proteinuria, haematuria, azotemia, or an active urine sediment with red or white cell casts. The serum C3 concentration is usually low and the C4 concentration normal; however, a normal serum C3 concentration does not exclude C3GN [10]. Immune complex glomerulonephritis, especially acute post-infectious GN, is the most common aetiology of CsGN in children [1, 3], but the serum complement concentration typically normalises within 6–8 weeks [5]. With these overlapping clinical presentations, it is not surprising that a subset of cases classified as atypical post-infectious glomerulonephritis are actually C3GN [5, 11]. It is also possible that some cases currently diagnosed as pauci-immune crescentic and necrotising GN may actually result from AP dysfunction and particularly the 15% of cases that are ANCA negative, which may have C3 staining dismissed as a nonspecific finding [11]. In the present case, a high clinical suspicion and prompt kidney biopsy performed after only 7 weeks proved quite useful in achieving an early and correct diagnosis.

After diagnosis, specific tests should be performed to help identify the underlying aetiology of C3 glomerulopathy and monitor disease progression, as this knowledge may help guide therapy. Most reports describe a diverse array of acquired and genetic abnormalities resulting in AP dysfunction [4, 10–12]. Acquired causes of C3 glomerulopathy include antibodies affecting AP inhibition, such as anti-complement factor H or factor I, or those stabilising the activation of C3 convertase, such as C3 nephritic factor (C3Nef). Among known genetic abnormalities, new mutations are increasingly being detected in complement genes, such as those encoding factor H, complement factor H-related (CFHR) protein CFH, factor B, factor I, and C3. Mixed acquired and genetic abnormalities can also occur as reported by Servais *et al*., who observed C3Nef abnormalities, factor H mutations, and CFHR5 mutations in all 12 patients in a case series of biopsy-proven C3GN [10]. The patient in the present case did not show evidence of any acquired cause, and no genetic mutations were detected. However, the persistently low serum C3 concentration at presentation and the decreased AP activity immediately after discontinuing cyclophosphamide treatment both indicate ongoing AP dysfunction. Thus, this case highlights the value of examining complement activity in cases with a high clinical suspicion of C3GN, evaluating the disease progression, and monitoring functional AP activity as an early biomarker to detect relapse.

Without an underlying cause, it is difficult to recommend a specific treatment for C3GN based on previous cases. The paucity of reliable data on treatment options stresses the importance of publishing these cases reports, even those with a relatively short follow-up period. In treating CsGN, the available data supports the use of intravenous methylprednisolone followed by a tapering course of oral prednisolone combined with either cyclophosphamide or mycophenolate mofetil. We administered glucocorticoids and cyclophosphamide, followed by micofenolate mofetil, which has thus far proven successful. The previous case series demonstrated the importance of immunosuppression, such as eculizumab, when the soluble membrane attack complex C5b-9 is markedly elevated, as in DDD [13], a scenario that was not present in the current case. As previously reported, the outcome of CsGN in children is poor owing to the delayed diagnosis (mean 2.47 months) from the first episode of asymptomatic gross haematuria to the initiation of immunosuppression treatment. By the time treatment is initiated, renal insufficiency is often already present, and fibrous tissue prevails within the crescents [1]. Nevertheless, recent data suggest that CsGN is a poor independent predictor, which also supports our aggressive immunosuppression therapeutic approach [14].

## Conclusion

Our case emphasises the efficacy of early intensive immunosuppressive treatment in a patient with C3GN presenting with a crescentic pattern and normal renal function at diagnosis. In addition to the previously reported cases, decreased AP functional activity and severely decreased serum C3 concentration may be promising biomarkers to identify patients with concomitant AP dysfunction early and may help prevent progression of renal disease. Clinicians and pathologists should be aware of this possible diagnosis in order to avoid misdiagnosis and delaying treatment, especially in paediatric cases that commonly present with asymptomatic gross haematuria. Prospective studies are needed to determine the efficacy of these biomarkers and of aggressive immunosuppressive treatment in the management of crescentic pattern associated with C3 glomerulopathy, even in cases without renal insufficiency.

## Consent

Written informed consent was obtained from the parents of the patient for publication of this Case Report and any accompanying images. A copy of the written consent is available for review by the Editor-in-Chief of this journal.

## Abbreviations

AP: Complement alternative pathway
C3GN: C3 glomerulonephritis
CsGN: Crescentic glomerulonephritis
DDD: Dense deposit disease.
